# Supplementary figures and images for: Interaction of Receptor-Binding Domain of the SARS-CoV-2 Omicron Variant with hACE2 and Actin
Source: Cells. 2024 Aug 7;13(16):1318. doi: 10.3390/cells13161318 (PMC11352305; doi:10.3390/cells13161318)

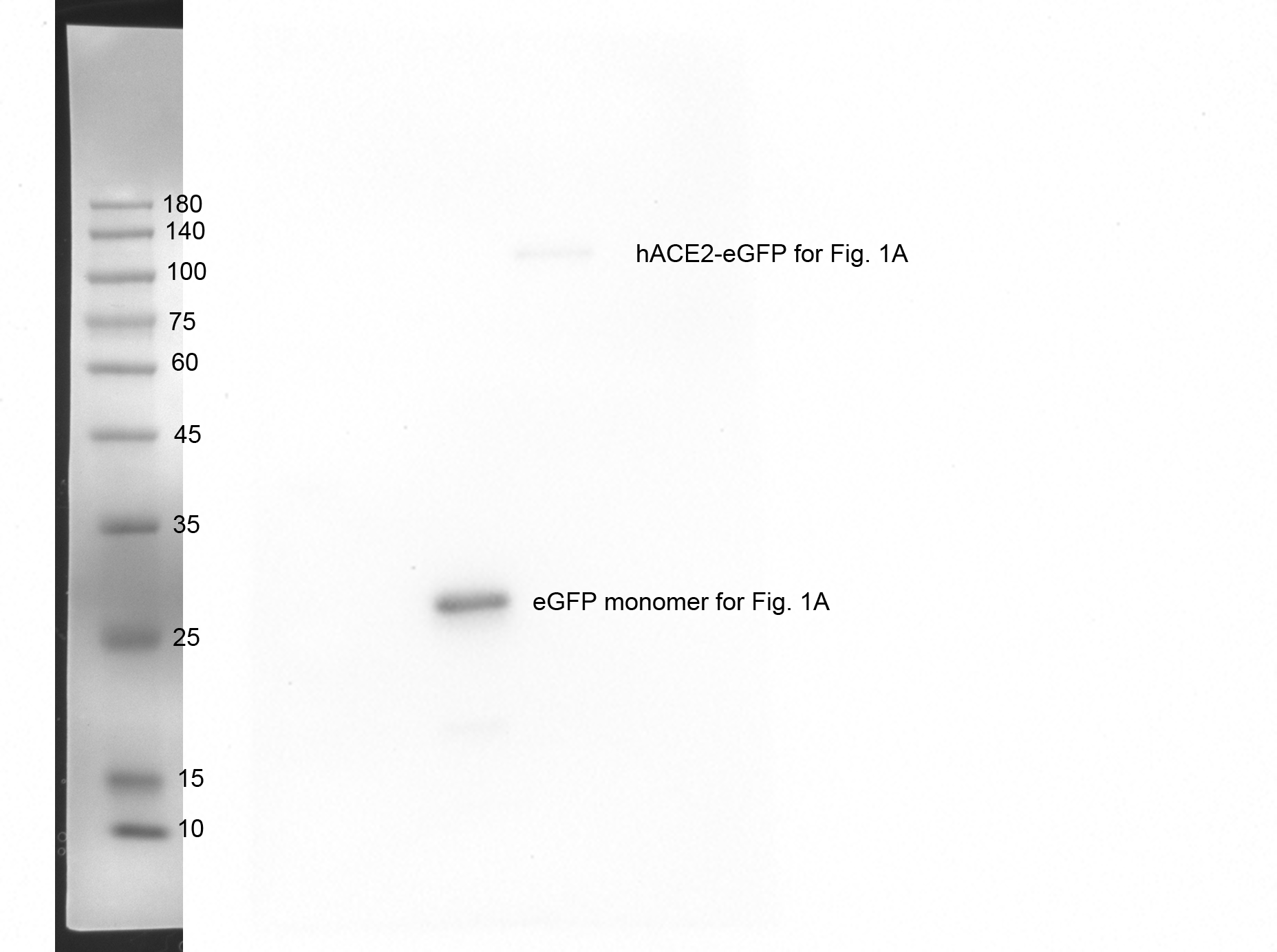

Supplement: Supplementary file 1 [file cells-13-01318-s001.zip › Figure S1.tif]

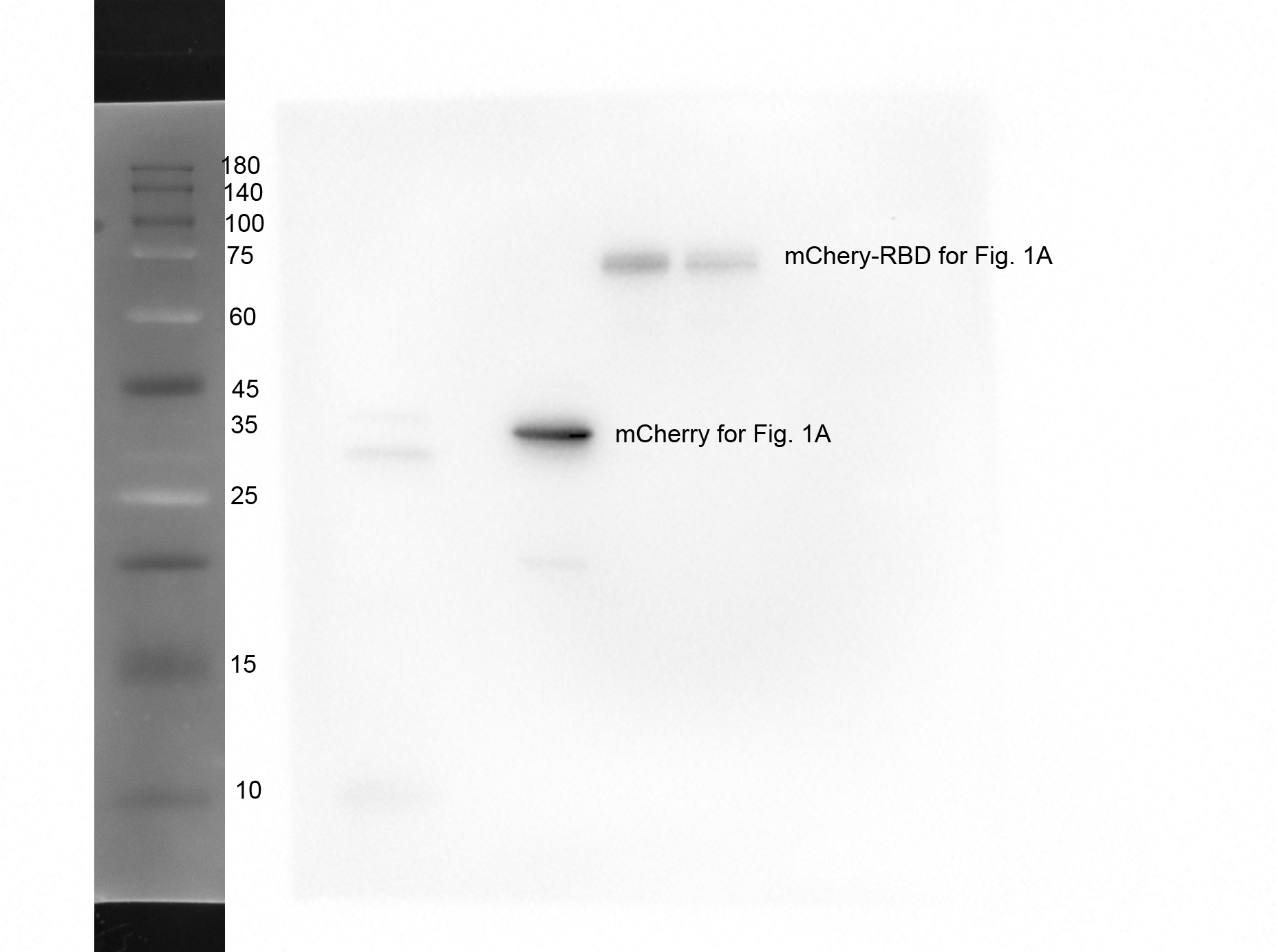

Supplement: Supplementary file 1 [file cells-13-01318-s001.zip › Figure S2.tif]

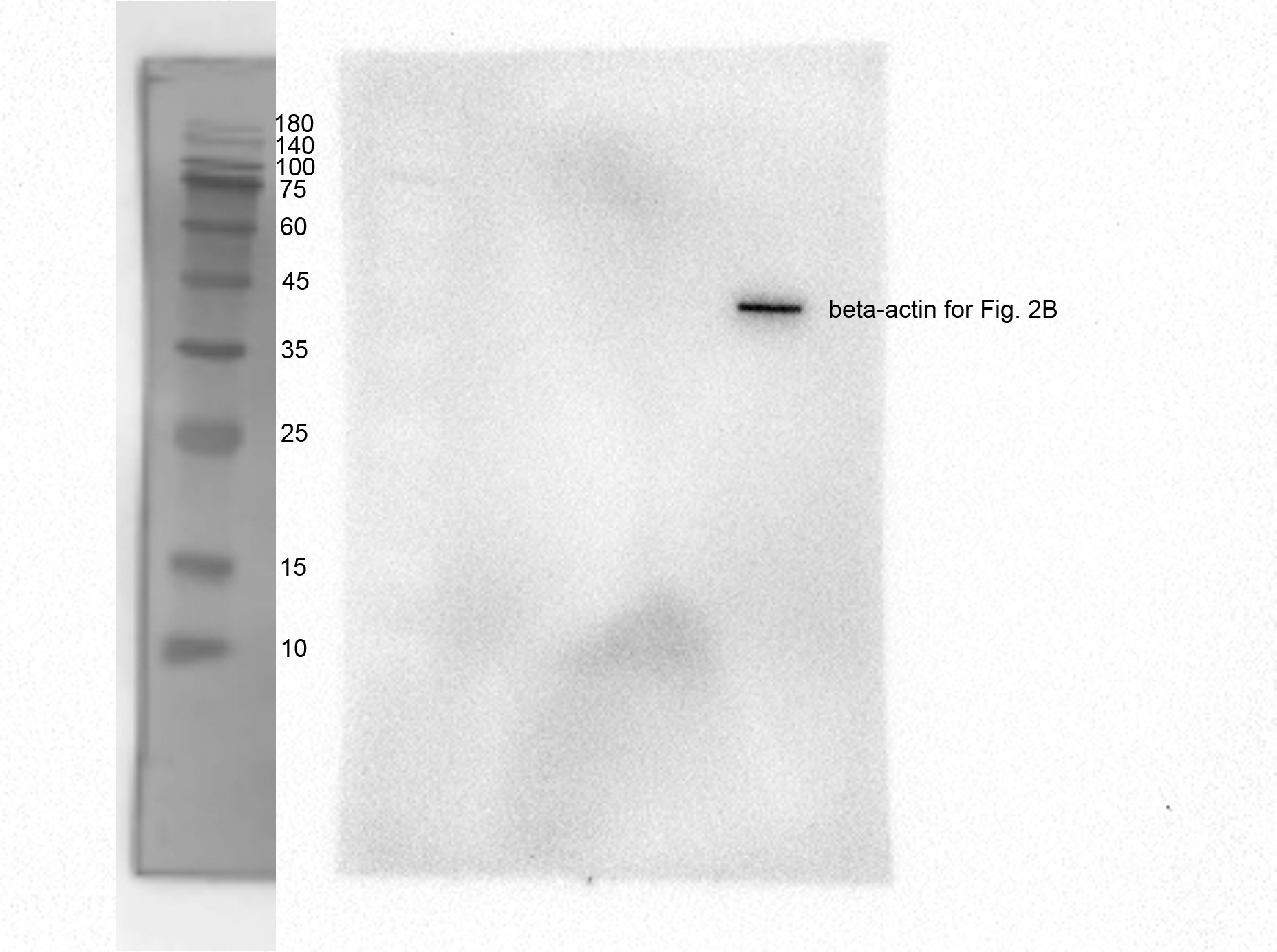

Supplement: Supplementary file 1 [file cells-13-01318-s001.zip › Figure S3.tif]

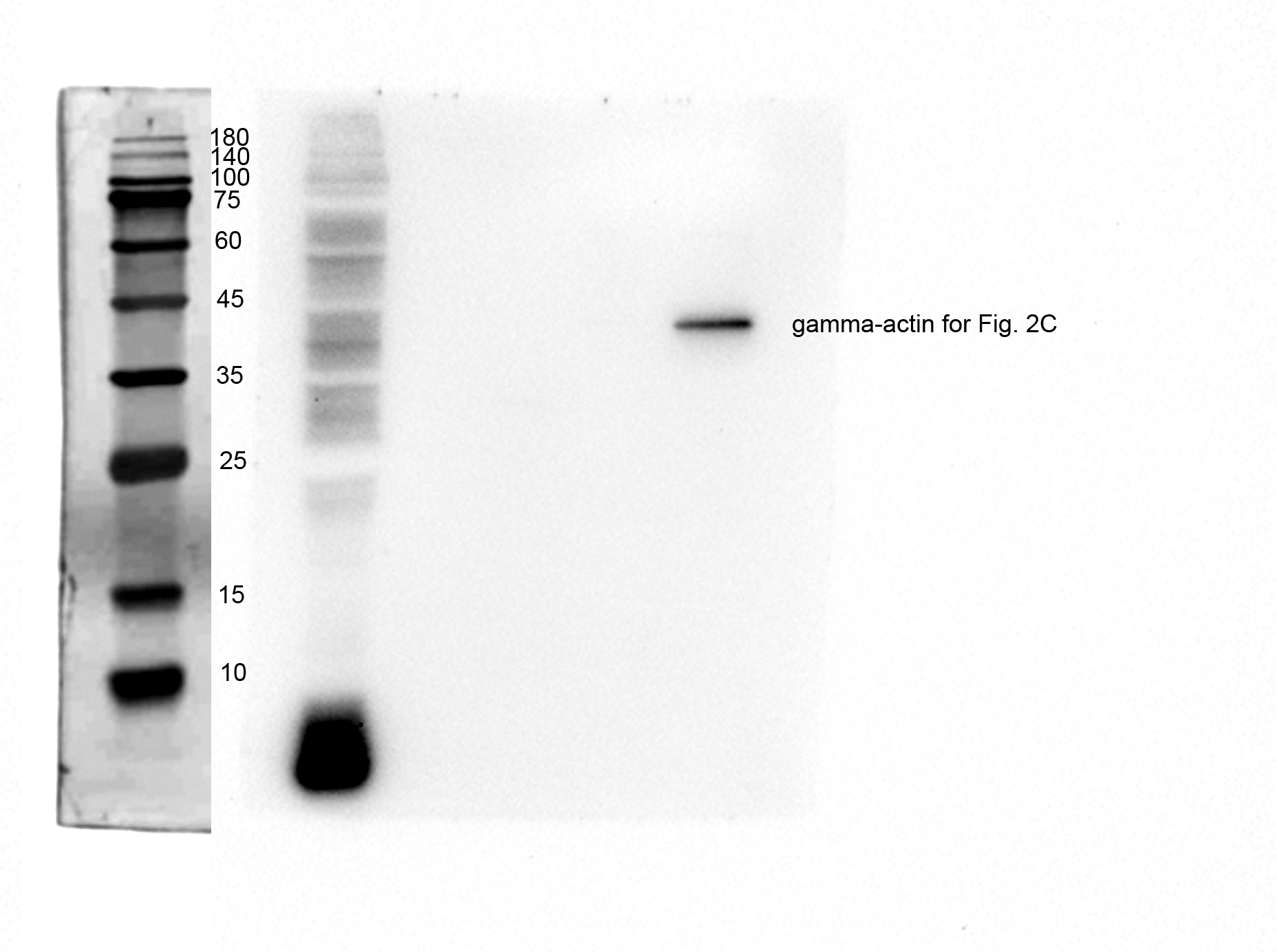

Supplement: Supplementary file 1 [file cells-13-01318-s001.zip › Figure S4.tif]
